# Electronic ordering and the management of treatment interdependencies: a qualitative study of paediatric chemotherapy

**DOI:** 10.1186/s12911-020-01212-z

**Published:** 2020-08-14

**Authors:** Valentina Lichtner, Bryony Dean Franklin, Luciano Dalla-Pozza, Johanna I. Westbrook

**Affiliations:** 1 Australian Institute of Health Innovation, Faculty of Medicine, Health and Human Sciences, Macquarie University, Sydney, Australia; 2 Department of Practice and Policy, UCL School of Pharmacy, University College London, BMA House, Entrance A, Tavistock Square, Bloomsbury, London, WC1H 9JP UK; 3 Centre for Medication Safety and Service Quality, Pharmacy Department, Imperial College Healthcare NHS Trust, London, UK; 4 Cancer Centre for Children, The Children’s Hospital at Westmead, Sydney, Australia

**Keywords:** Health information technology, Evaluation, Medication safety, System safety, Paediatrics, Hospitals, Human factors, Qualitative research

## Abstract

**Background:**

There are serious safety risks associated with chemotherapy, often associated with interdependencies in regimens administered over months or years. Various strategies are used to manage these risks. Computerized provider order entry (CPOE) systems are also implemented to improve medication safety. Little is known regarding the effect of CPOE on how clinicians manage chemotherapy interdependencies and their associated safety strategies.

**Methods:**

We conducted a multi-method qualitative study in a paediatric hospital. We analysed 827 oncology incidents reported following CPOE implementation and carried out semi-structured interviews with doctors (*n* = 10), nurses (*n* = 6), a pharmacist, and oncology CPOE team members (*n* = 2). Results were interpreted according to safety models (ultra-safe, high-reliability organisations [HROs], or ultra-adaptive).

**Results:**

Incident reports highlighted two interrelated types of interdependencies: those within organisation of clinical activities and those inherent in chemotherapy regimens. Clinicians reported strategies to address chemotherapy risks and interdependencies. These included rigid rules and ‘no go’ contexts for treatment to proceed, typical of the ultra-safe model; use of time (e.g. planning only so far ahead) and sensitivity to operations, typical of HROs. We identified three different time horizons in CPOE use in relation to patients’ treatments: life-long, the whole regimen, and the ‘here and now’. CPOE supported ultra-safe strategies through automation and access to rules/standardisation, but also created difficulties and contributed to incidents. It supported the ‘here and now’ better than a life-long or whole regimen view of a patient treatment. Sensitivity to operations was essential to anticipate and resolve uncertainties, hazards, CPOE limitations, and mismatches between CPOE processes and workflow in practice.

**Conclusions:**

Within oncology, CPOE appears to move the ‘mix’ of risk strategies towards ultra-safe models of safety and protocol-mandated care. However, in order to operate ultra-safe strategies embedded in CPOE and stay on protocol it is essential for clinicians to be thoughtful and show sensitivity to operations in CPOE use. CPOE design can be advanced by better consideration of mechanisms to support interdependencies.

## Background

Chemotherapy medications are hazardous, high risk treatments; errors with these medications can cause severe harm, especially in children [1]. Chemotherapy regimens are administered often in combination with other medications and over many months, and errors are often associated with interdependent elements of chemotherapy regimens, such as number of cycles, dose scheduling, cumulative doses, and monitoring [1, 2].

To reduce harm from chemotherapy, risk management strategies must be in place [3], individual patient regimens must be based on documented and referenced protocols [4], and work processes organised with reference to accepted standards [4–7] and designed to reduce the possibility of error [8, 9]. Computerized provider order entry (CPOE) systems with decision support are a further strategy for improving safety with chemotherapy. CPOE can reduce medication errors and unwanted protocol deviations by automatically calculating chemotherapy doses based on patient height and weight, providing warnings, assisting in scheduling of chemotherapy cycles, and supporting workflow [10–16]. Conversely, CPOE can introduce new types of error and have unexpected negative consequences on work practices [1, 17]. It can also be challenging to implement CPOE for chemotherapy [15, 16, 18, 19], with every chemotherapy protocol having to be entered in the system and then kept up to date [15, 20]. Adoption of CPOE for chemotherapy can thus lag behind adoption of CPOE systems for other medications or in other clinical areas [15].

The literature suggests that design of CPOE for chemotherapy is ‘confronted with problems’ [21] and that CPOE alone is not sufficient to eliminate chemotherapy errors. Other safety strategies generally co-exist, including error surveillance systems [22, 23], checking of patient regimens against standard protocols [22, 24], and interventions to improve situational awareness [12, 22]. The literature remains unclear on how CPOE relates to such strategies, especially with regard to management of interdependencies. This gap in knowledge may hinder efforts to improve CPOE design and implementation, and thus improve patient safety.

This study aimed to fill this gap by investigating how CPOE for chemotherapy relates to other safety strategies in use in a paediatric clinical oncology unit, with a focus on the management of interdependencies. Our objectives were to identify the strategies clinicians apply to safely manage the interdependencies inherent in paediatric chemotherapy, and whether/how CPOE affects these.

For this study, we drew on theoretical and practical insights from Vincent and Amalberti’s research on safety strategies ‘in the real world’, and the three main safety models they propose - ultra-safe, high-reliability organisations (HROs) and ultra-adaptive [3, 25, 26]. We briefly explain their framework in the next section, as we describe our methods and provide definitions.

## Methods

### Analytical framework

**Fig. 1:**
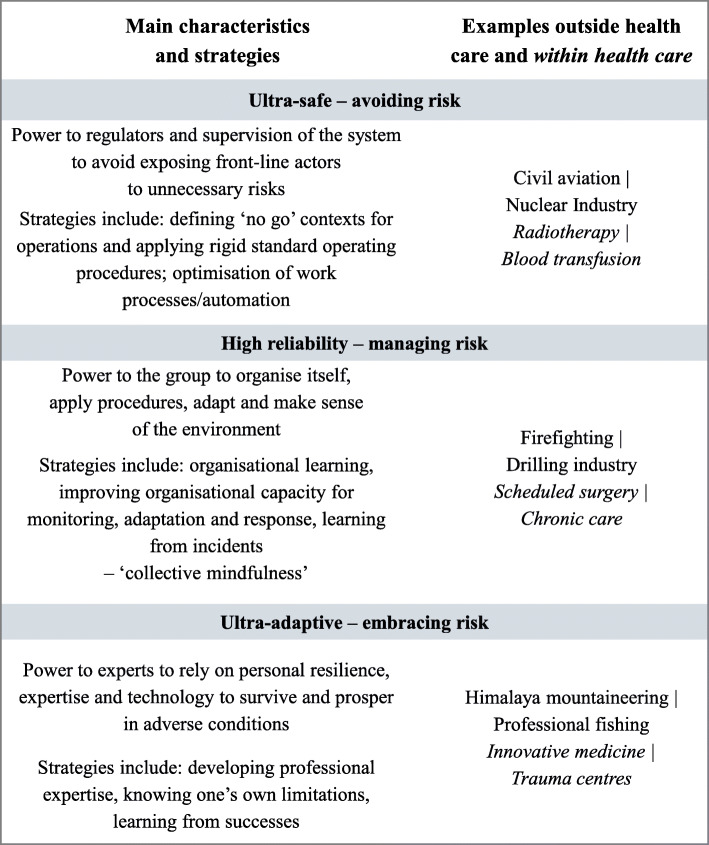
Three models of safety and examples of associated risk strategies. *Elaboration of Vincent and Amalberti text and figures* [3, 25]*, with additional reference to collective mindfulness – typical of high reliability organisations models of safety* [27]*. Collective mindfulness manifests when people on the frontline collectively show preoccupation with failure (ongoing wariness that errors are possible), reluctance to simplify interpretations of unexpected events (questioning assumptions, uncovering blind spots), sensitivity to operations (having an integrated understanding of current situation,* e.g. *awareness of how one’s actions affect others), commitment to resilience (awareness that it is impossible to anticipate all situations, needs for adaptation) and deference to expertise (persons with expertise to make decisions regardless of hierarchy)* [27]

Vincent and Amalberti [3] explain the different ‘real world’ safety strategies applied in different sectors or organisations for dealing with risks. Such strategies may involve, for example: 1. eliminating exposure to risk by defining ‘no go’ contexts for operations (following Amalberti, we refer to this as ‘Plan A’ [25] – in aviation, equivalent to grounding all flights when a volcano erupts) and applying rigid standard operating procedures; 2. introducing barriers to risks by engineering optimisation of work processes (including by introducing technology); 3. dealing with risks by improving organisational capacity for monitoring, adaptation and response (e.g. by maintaining ‘sensitivity to operations’ [27], or awareness of how one’s actions affect others); and 4. developing professional expertise for extreme situations. We considered these strategies as different ways, at different levels, to manage risks associated with interdependencies. These strategies also approximately correspond to three models of safety in organisations (Fig. 1)– the ultra-safe (examples 1 and 2 above), high reliability organisation (HRO) (example 3) and ultra-adaptive (example 4) [3, 25]. Different healthcare contexts have a different combination of the three models, with some working more often than others as ultra-safe, HRO, and/or ultra-adaptive, along a continuum. Thus, it may be expected that: *‘A healthcare team might, in one afternoon, work in an ultra-safe manner at some points, such as when a care pathway is clearly defined and entirely appropriate for the patient; they may work in a high-reliability mode for the main part and, for short periods, in an ultra-adaptive mode.’* [3]

Building on Thompson’s Organization in Action [28], we define *interdependency* as the relation between tasks or activities, where related tasks/activities must all be completed in a timely fashion, reliably, and/or safely, to achieve safe processes and/or outcomes. While many interdependencies are *sequential* (one task contributing to the next; a task cannot be completed unless other tasks have been completed first), others may be *reciprocal* (one task contributing to the other and vice-versa), or *pooled* (each activity independently contributing to the whole) [28]. For example, nurses’ chemotherapy administration depends on doctors’ prescribing, which may depend on pathologists’ reporting results (sequential tasks); supplying medications in hospital pharmacies on a just-in-case basis, contributes to doctors prescribing medications to patients, while doctors prescribing also calls for medications being stored in pharmacy on a just-in-case basis (reciprocal relationship between activities); repeated administration of chemotherapy doses to a patient cumulates towards a safe maximum level (pooled activities). We define *time dependencies* as those where safety in management of the interdependency requires attention to timeliness or timing of related tasks (e.g. administration of sequential doses at specific time intervals).

### Setting and design

The study took place in the oncology unit of a 350-bed tertiary paediatric hospital in New South Wales (Australia) between September 2018 and June 2019. The oncology unit is the largest children’s cancer unit in the State, with up to 150 new referrals each year. The unit has an inpatient ward plus an outpatient clinic run as an ‘open door’ service where patients and their families can be seen without an appointment. Doctors are routinely called to see patients not under their regular care.

Eighteen months prior to this study, the hospital had implemented hospital-wide CPOE (Cerner [29]) for prescribing and administration of medications, test orders and results, as part of an electronic medical record system. The oncology unit made use of CPOE functionality for linking orders (‘PowerPlan’) to incorporate chemotherapy regimens into the system and to prescribe and administer chemotherapy for individual patients. A chemotherapy PowerPlan team was responsible for building chemotherapy protocol templates in the CPOE and training oncology clinicians in use of the system.

We carried out a qualitative, interpretative study combining incident report analysis and interviews. Analysis of the incident reports informed the interview questions, and the insights gained through the interviews fed back to further analysis of the events described in the incident reports.

### Data collection

Incident reports were submitted by staff through the hospital online incident reporting system. Any event that resulted in (or had potential to lead to) injury, damage or other loss is required to be reported. The hospital provided us with all incident reports concerning oncology patients recorded in the period 15 August 2016 to 15 February 2018 (the first 18 months following CPOE implementation in oncology), whether or not these were specifically related to medications or CPOE.

Interviews were carried out by a researcher (VL) with a background in health informatics and qualitative research. All clinicians in the oncology unit and members of the chemotherapy PowerPlan team were invited to participate as we were seeking to maximise variety of levels of seniority and CPOE expertise. Access to participants was facilitated by the unit coordinator. We sought participants for interviews until we reached code and meaning saturation [30]. Interviews were semi-structured, using questions (Additional file 1) aimed to uncover how clinicians deal with interdependencies in the medication process, and whether the CPOE supported their work. With participants’ written consent, interviews were audio recorded and professionally transcribed.

### Data analysis

We carried out a qualitative content analysis [31] of the text narratives of the incident reports (fields for *Incident description* and *Contributing factors)* and the interview transcripts. Analysis was carried out with support of NVivo v.11, by one researcher in discussion with co-investigators. The free text fields from incident reports were treated as narrative accounts of events from frontline clinicians [32, 33]. All reports were included, but only those related to direct patient care coded. We took both a conventional (inductive) content analysis approach, with open codes derived from the data, and a directed (deductive) approach with our objectives providing higher level categories for grouping the open codes.

Analysis of interview transcripts was carried out iteratively. First, an in-depth detailed understanding of the data was gained through careful reading and line by line coding. Open codes were applied through both conventional and directed approaches, to address study objectives and capture significant aspects of the context. Chemotherapy medication processes, as described by clinicians in the unit, were represented in flowcharts to better understand interdependencies in workflow. This analysis was done concurrently with, and soon after, data collection. A framework analysis approach [34] was then applied using categories from Vincent and Amalberti’s model.

Throughout the analysis process, discrepant findings were sought. Findings from incident analysis were triangulated with those from interviews to enrich understanding [35]. The head of the oncology unit (LDP) checked ‘the “fit” between respondents’ views and the researcher’s representation of them’ [36] (credibility).

## Results

A dataset of 827 incidents were received from the hospital and included in the analysis. Incidents not concerning direct patient care were then excluded (*n* = 73). (Details of the 827 incidents are given in Additional file 2 and elsewhere [37]). In the analysis of the incidents, the CPOE appeared to be both a mechanism for safety and a contributing factor for incidents.

Twenty interviews were conducted with 19 participants (Table 1). Interviews lasted about 30 min.

**Table 1:** Interview participants

Role	n
Consultant oncologists ^a^	2
Fellows	4
Registrars	3
Senior resident medical officer	1
Specialist pharmacist	1
Nurse unit manager ^b^	1
Registered nurses ^b^	4
Clinical nurse consultant	1
Information technology team - oncology pharmacist background	1
Information technology team - oncology nurse background	1
Total	19

^a^ one interviewed twice; ^b^ within the same group interview. Paediatric oncology fellows and registrars in New South Wales (Australia), are resident doctors with oncology as their speciality, considered ‘consultants in waiting’ and ‘consultants in training’ respectively. This notation may be different internationally

We integrated results from interviews and incidents analysis and structured these around interdependencies and risks to medication safety, strategies to deal with these risks, and the role of the CPOE for each strategy. We identified quotes from interview transcripts by interview number (idX), but do not provide details on interviewees’ roles to protect their anonymity. We refer to incident reports by their row in the dataset (iX).

### Interdependencies and risks to safety with chemotherapy

Work practices related to paediatric chemotherapy treatments were rich in interdependencies. Across interviews and incidents, we identified two interrelated overarching types of interdependencies that characterised chemotherapy prescribing and administration: first, those related to the organisation of clinical activities, in particular the medication workflow involving doctors, nurses and pharmacy staff, and its interplay with a variety of hospital services (e.g. intensive care, pathology, imaging, as well as other hospitals), and second, interdependencies inherent to chemotherapy regimens that dictated combinations of medications and tests. Time dependencies were especially apparent, such as precise time gaps between administration of successive doses.

Both types of interdependencies (organisational and regimen-related) were identified in incident reports and often involved CPOE. For example, an incorrect date in a CPOE prescription and a delay in documentation of the patient being ‘ready for chemotherapy’ (organisational dependency-medication workflow) delayed administration due to the medication requiring pre-medication (a regimen dependency), which further cascaded into requiring monitoring of the patient ‘after hours’, leading to increased risks for the patient and costs to the hospital (organisational dependency).

*[...] Patient due rituximab and MTX [methotrexate] today, date for chemotherapy was incorrect and not ‘ready for chemotherapy’ documented [in the CPOE]. This delayed chemotherapy administration until 11am. [...] [medications] will need to be reordered. The first medication requires premedication and the patient is at risk of anaphylaxis with this drug (needs to be given in business hours) and MTX requires blood monitoring levels and if given outside set hours adds significant cost [...] [i150]*

### Ultra-safe risk strategies applied to chemotherapy processes

Chemotherapy safety risks were reported to be managed through a range of strategies based on the application of rules and multiple safety checks. These appeared more typical of the ultra-safe model of safety [3] than the HRO model.

Specifically, participants spoke of rigid rules regarding who was authorised to prescribe chemotherapy (on CPOE) and the content of medication orders as per chemotherapy protocols. Continuing with a regimen (e.g. progressing to the next cycle) was organised around the principle of withholding treatment until the patient recovers (comparable to ‘Plan A’ in the ultra-safe model).

Participants reported multiple checks during the medication process. In particular, nurses were tasked with checking each dose against the protocol to alert doctors to any discrepancy. This was ‘*an institution specific practice’* (id16) that was maintained after CPOE implementation, despite the use of pre-set CPOE templates that would (or should) support doctors to produce orders matching the corresponding protocol.

*...when I am ordering it [on CPOE], it looks fine to me. [But] The nurses [would] say, “Well, can’t do that because it is out of sequence,” or, “you have pushed this onto that.” [...] Or they will say, “The protocol mandates 120% of this and you are giving 110% or 150%.” And they are just simply saying, “Is this what you want me to do?” ... (id16)*

Sometimes the patient’s condition led to clinicians deciding to deviate from the protocol – such as when ‘*this child has such a high-risk disease that no matter what I’m going to go ahead’ (id16)* despite test results not reaching the threshold indicated by the protocol. This kind of judgement – highly dependent on individual clinical expertise - typically belongs to the ultra-adaptive model of safety. However, in this context – where the doctor had to communicate the decision to the nurses and to other doctors in order to act on it – it is perhaps suggestive of a HRO approach to dealing with risks.

### Does CPOE support an ultra-safe model of safety with chemotherapy?

CPOE supported strategies typical of the ultra-safe model through hard and soft mechanisms, namely through automation, access to information and standardisation of the semantics of protocols (disambiguation) (Table 2).

**Table 2:** Ultra-safe strategies to avoid medication errors/unwanted protocols’ variations: automation and ‘soft’ support

Risk strategy (ultra-safe model)	CPOE support	Examples from the data of how the CPOE supports the use of ultra-safe strategies (compared to paper-based systems)
Treatment is independent of specific clinicians	Visibility and access to protocol rules and a patient prescribed treatment	*[...] your treatment has to go ahead whether I’m alive, dead, overseas [...] someone else has to bear the brunt of knowing where they’re at. So it’s really crucial for us to have a system whereby the – ‘where they’re at’ is easily accessible, (id1)*
Everyone knows/has access to the rules	Visibility and access to protocol rules and a patient prescribed treatment	*...we have got immediate access to what the protocol looks like and [...] to the documentation. [...] they’re not squirreled away in some cupboard, they’re not lost, they’re available electronic everywhere. (id1)* *[nurses would] go to the orders page. [...] And they’ll go okay, so he’s in hospital [...], so he came in on Sunday, tomorrow he’s due vincristine. I have to make sure I check with the doctors, whether they want him to go ahead with it or delay it, which they would never have known in advance, previously. They’d never have been able to see that. Because they didn’t have any of their chemotherapy records when they got admitted. [...] Their visibility of it is probably one of the most important things of the plans. (id14)*
Standardised protocols across patients and clinicians	Disambiguation of protocol rules	*… whether an or and a comma in a sentence meant one thing was inclusive of another, or one thing was exclusive of another. So even within protocols, even the smallest wording, [...] you’ve really got to sit down and discuss as a team what you think it means, … (id13)*
Rules on the content of the prescription (as per protocol)	CPOE templates, with linked pre-set orders Automatic calculations by embedded rules Warnings about breaking rules	*...depending on the drugs that go into [the CPOE plans] and how they relate to each other [...] they might [...] have a timed relationship to say you’re not allowed to start drug Y until drug X has finished or run them both at the same time... (id13)* *You’re meant to give this over four hours, [...] - in paper, that was just written and then pharmacy would come along and, kind of, annotate it, whereas now it’s prebuilt ... (id7)* *all the drugs listed here, you just have to agree to it [...] [the CPOE system] will limit dose, [system says two] you can’t order two and a half. So there are certain protections in the system... (id1)*
Rules on who can act on the prescription	Users’ profiles and workflow management systems to route the prescription	*... [the fellows] – they can sign it but it doesn’t go to pharmacy until it’s [gone] to the oncologist. [...] Once [the oncologist] reviewed it, it goes to pharmacy. [...] [Pharmacy] can’t make it up until [it’s been reviewed by the oncologist]. (id1)*
Treatment stopped until patient recovers [‘Plan A’]	‘Ready for chemo’ checkbox – no administration until ‘ticked’	*...there is a flow that says, well say it’s more than 30 white cells, [...], liver function is less than that, [...] very clear criteria and then only if you tick them and if they are okay will it allow you to go ahead and sign for a go ahead. (id2)*
Checks along the process	Double signatures required to unlock next step	*...we are much more rigid in terms of two people checking, two people going to the bedside. (id8–12)*
Checks against the rules	Nurses verifying treatment based on access to the protocol	*... [before CPOE] you had to have the paper protocol [...] [with the CPOE] the nurses [...] [have] access to the protocol. So, when they saw a particular dose scheduled, they would go to the protocol, check it and say ‘yes’. And, [...] if it didn’t marry, then they would report it as an incident ... (id16)*

In terms of automation, the CPOE provided clinicians with all required medications pre-built in templates, to reduce the possibility of medications being omitted by mistake, which were also linked with appropriate time dependencies (if doctors delayed one, the others would be automatically delayed for a corresponding period). The CPOE provided automatic dose calculations and warnings based on embedded rules, and ‘exposed’ unreliable patient weight and height data used for dose calculation, by displaying trends and normal ranges.

*... you can see patients with a weight here and a height up here [on the CPOE chart] [...] three months for a baby of four kilos you can go to eight kilos, you can change dramatically the dose. [...]. Now, in paper land [...] [doctors] might go six months without checking the height and weight. It happened before, but here it’s exposing it. (id1)*

CPOE automation also limited prescribing and administration rights through different users’ profiles; the embedded workflow management system automatically routed the orders to the authorised person. The CPOE paused the workflow proceeding forward to administration until the system had been notified via a checkbox that the patient was ‘ready for chemo’.

However, these CPOE contributions towards an ultra-safe approach to chemotherapy were challenged by the perceived complexity of operating the system (especially when protocol templates were not available in the system to prescribe a patient’s regimen), and difficulties in identifying ‘where the patient is at’ in the protocol.

In terms of ‘soft’ mechanisms, we identified two aspects of CPOE that supported use of ultra-safe risk strategies. All clinicians had access both to patient information and the relevant chemotherapy protocol; previously, paper-based protocols were kept in drawers and not easily accessible across place and time. In addition, the process of converting paper-based protocols into electronic versions brought to the surface previously invisible ambiguities in their rules. To automate these rules, these had to be clarified and uniquely defined so that all clinicians would interpret the protocol in the same way.

### HRO strategies - management of uncertainties and ‘usual hazards’ through adaptation and different time horizons

The application of the principle of withholding treatment until the patient recovers (‘plan A’) meant that planned doses or cycles often needed to be rescheduled. Thus, for any patient there would be uncertainty about when exactly the treatment would be given. This scheduling – or rescheduling - of the treatment must also take into account the constraints posed by the hospital’s resources (organisational interdependencies), for example ‘*our general anaesthetic days are Monday and Thursday’* (id16), or over holidays, ‘*services aren’t working quite as well’* (id1). The patient and family may also pose constraints on suitable dates – such as wanting to avoid school photo day.

Clinicians reported that it was challenging to plan cycles, tests and doses much in advance. Although the CPOE theoretically allowed clinicians to prescribe a whole regimen in advance, given these uncertainties, interviewees indicated that they would only schedule about a month ahead in practice.

*Researcher: the doctors might schedule the entire one year of treatment in advance? Participant: [...] they probably wouldn’t. [...] we would chart chemotherapy, kind of, a month out, because [children] can grow so much in such a short time, the doses need to change. Other things can happen as well that they may start to not recover as well, so we need to modify the dose, it was too big for them, so we need to back off [...]. (id7)*

Doctors therefore made use of time as a risk management strategy [25] to deal with the uncertainties. More specifically, we identified three time horizons used to approach a patient’s chemotherapy regimen – a life-long view, the whole regimen view, and the ‘here and now’. CPOE automation better supported the ‘here and now’ than the longer time views (Table 3). For example, clinicians reported difficulties with finding and collating information to calculate a patient cumulative dose (a life-long view), and with the fragmented electronic display of the whole regimen.

**Table 3:** Doctors’ time horizons over chemotherapy treatments and computerised provider order entry (CPOE) support

Time horizon^a^	Uncertainty and/or risks associated with interdependencies managed with this time horizon	Examples of how CPOE supports this (or not)*	Examples from the data
life-long	A view over cumulative effects of medications over time, to manage life-long risks (e.g. hearth failure)	Compared to paper-based records, CPOE makes patient information available, independent of time and place (*) Difficulty in tracing specific data across different orders/screens (*) IT teams can manually build into the system automated alerts on cumulative doses – but this must be done for each drug, and may not be available when needed	*...the cardiologist team always need to know how much anthracyclines they’ve received, because it’s cardiotoxic and the dose that they’ve received makes a difference about what was risk stratifying and things. So to try and find a patient’s cumulative anthracycline dose sometimes has been exhausting. More exhausting than night shifts exhausting. (id20)*
whole treatment	A view over ‘where we are at’ in the protocol as a whole; how the protocol is being given – possibly adapted – to the patient, and the patient response to treatment. It is a view of how much chemotherapy overall is needed, to minimise toxicity and maximise effect, and how it is given over time	Compared to paper-based records, CPOE makes patient information available, independent of time and place (*) Lack of a summary overview/graphic display of the whole regimen with any variations of protocols applied to the patient in the past and planned future doses (*) Information is available but very detailed and fragmented	*I can go through a three-day admission, it’s all there in a capsule. But when that becomes 6 months or 12 months or 5 years, ... the system is not geared to allow us to navigate to the critical information [...] The treatment is listed as a series of lines [...] it’s very hard to actually visualise that they haven’t got an extra dose at day 15 or they’ve missed a dose at day seven, ... (id6)*
here and now	A view over the requirements and constraints for the administration of the current dose, to prevent medication errors, unwanted deviations from protocols, and delays	Automated pre-coded prescription items, time dependencies and scheduling, all items included in the prescription at planning stage	*...the main thing is the system is built on protocol. [...] it follows almost exactly the way the protocol is written, almost all the time. And if in case what I am prescribing is a deviation from what is allowed, then [...] it is going to flag up. For example, if something that I needed to be charted for day 1, I charted again for day 2 and 3 [..], then it will say, it’s already there why are you [ordering] it? [...] and it’s really handy that [...] the cut offs [safety thresholds] are already there, right next to that. (id2)* *the timing [...] often it’s pre-suggested as well, depending on what medication you’re charting it will already have a suggested frequency that you should be charting it (d5)*

^a^ Over a *life-long view*, there are concerns for life-long risks of cumulative doses. *A whole treatment view* is required for most treatment decisions; each dose is assessed in view of patient’s response to previous doses, in relation to future doses, future options for treatment - at any point in time, the sensemaking is retrospective and prospective. The *view over the here and now* is concerned with ‘*the minutiae’* of the medication process related to the specific dose, around the time of administration. It’s where the order is finalised, multiple checks performed, medications given (or not) – a time that may span over a few days

### Sensitivity to operations and CPOE

**Fig. 2:**
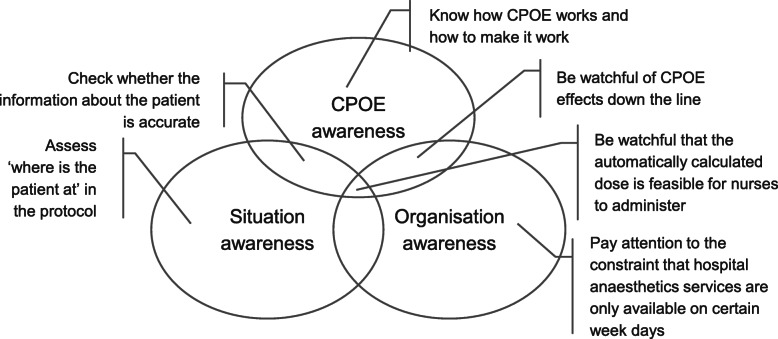
Sensitivity to operations in chemotherapy prescribing (with examples). CPOE = Computerized Provider Order Entry system. We refer to Endsley’s definition of situation awareness [38] as “the perception of the elements in the environment within a volume of time and space, the comprehension of their meaning and the projection of their status in the near future”, applied to a patient. With organisation awareness we refer to the understanding of how roles and services fit within the organisational structure, institutional practices and rules, and how to operate within these practices to achieve desired effects. This definition includes individual awareness of local teams and workflows, or of the wider hospital organisation, within or beyond individual’s control. CPOE awareness is knowledge on how the technology works/how to make it work/the effects it produces and acting on this knowledge. We take sensitivity to operations (at individual or collective level) as any and all of the three types of awareness at any one time

We found that a combination of situation awareness, organisational awareness and CPOE awareness was perceived as essential for safe and efficient CPOE use (Fig. 2). Clinicians had to maintain (and act on) situational awareness to understand ‘where is the patient at’ in their specific regimen and with respect to the corresponding protocol. Several interviewees referred to this information need – ‘where patients are up to’ (id1, id5, id7, id8–12). Clinicians also had to maintain (and act on) organisational awareness. For example, they had to act on the system with awareness of self and others ‘down the line’ (Table 4). They had to be aware of CPOE, learning to be watchful of its automated behaviour such as automated recording of times and dates. With use, clinicians learned that, for a variety of reasons, time and dates in the system may not accurately reflect the times when medication activities took place. This had repercussions for regimen time dependencies, which were also encoded in the templates.

*...sometimes when [the CPOE] says, it’s the start date, it’s not really been the start date, because it’s been delayed [...]. That stays as the estimated start date [...] when you come to [prescribe], they’re due next week, for example, but if you look at the chemotherapy, [...], they’re due in two weeks. [...] just [automatically] ticks over [...] (id3)*

**Table 4:** Examples of (organisation) team level awareness and computerised provider order entry (CPOE) limitations

Team perspective	CPOE issue to be aware of	Examples from the data of awareness of the impact of the CPOE for the team
Doctors with doctors	Difficult to identify ‘where the patient is at’ [whole regimen view]	*....you do have to then plough through a fair bit of information to figure out where they’re at. So I’m at the mercy of the quality of the input of the previous doctor [...] If they put in [...] for example, “had day 72 coming on the 14th [of this month] for day 86,” then it places me exactly where they’re at. [...] if they put that in it makes my life easy. If they put in, “See again next scheduled visit”, I don’t know what that next scheduled visit is. And for my patients I might know, [...] I will see other people’s patients [...] I have to then come up to speed with what that particular patient is on. (id1)*
	Difficult to identify [patient specific] changes to prescriptions	*I know the things that make my life easier, so I will document these things whenever I see them. And some of the fellows that see them in clinic, because they were more recently registrars and recognise this issue, will do the same. So they will document clearly their medication list, and the changes that they’ve made. (id20)*
Doctors with nurses	Difficult to identify ‘where the patient is at’ [exact place in the protocol (a PDF file)]	*Nurse 1: [...] when the fellows are ordering the chemo [...] it does help if they are actually very specific on where this kid is up to, like, exactly what cycle and everything they are up to.* *Nurse 2: ‘Course two, cycle three, page 59’.* *Nurse 1: The more specific they [doctors] are, the easier it is for us to find it, yeah. (id8–12)*
	Automatically calculated doses may not be feasible to administer	*[as a doctor] I make sure that the dose isn’t something that’s ridiculous and going to be really hard for [nurses] to give. (id5)*
	Automatically calculated times of doses may not be convenient (safe?)	*I make sure that [nurses] can recognise if they need to give it now or if they can give it at a later date, like [...] the midnight doses. [...] whether the times that are coming up are convenient. (id5)*
	Fluid charts may not get automatically filled-in	*... just things like being aware that if they [the doctors] don’t fill out things like durations and infuse over times correctly, it makes it much harder for the nurses to then complete their fluid balance charts correctly, because they’ve got to manually pull everything in, whereas if the doctors order it correctly it all prepopulates beautifully for them. (id13)*
Nurses with nurses	Difficult to identify ‘where the patient is at’ [exact place in the protocol (a PDF file)]	*Nurse 1: To find the bit you actually want for the chemo you are giving, you might have to scroll through multiple pages if you are the first [nurse] to kind of ... find that cycle of chemo.* *Nurse 2: Once you found it we write it down [for the next nurse]. (id8–12)*
Doctors with Pharmacy	Automatically calculated doses may not be feasible to dispense	*So not prescribing a dose that’s 1.62 mg because no pharmacist is ever going to be able to draw that up ... (id13)*
	Pharmacy staff access to CPOE orders not sufficient for timely dispensing	*We owe it to the pharmacy to give them as much notice as possible and the minimum [lead] time we have most recently put in is 48 h. So the pharmacy has to know at least 48 h before, we’re going to give the drug... (id2)*

## Discussion

We studied a paediatric oncology unit using a hospital-wide CPOE system, and identified two inter-related overarching types of interdependencies associated with chemotherapy: those related to the organisation of clinical activities, and those inherent to chemotherapy regimens in terms of dictating precise combinations of medications, and combinations of medications and tests. Time dependencies were especially apparent across both interdependency types. The time dependencies in a patient regimen were dealt with through a cognitive strategy known as ‘fragmentation’ (‘allowing shorter horizon planning’ [39]) and a practice of temporary plans and constant adjustments. These we associate with ‘adaptation’ strategies typical of HRO models of safety.

CPOE automation seemed to better support the small scale, short-time regimen dependencies that are largely under control of the team, rather than the scheduling of services where organisational dependencies are not under their control. It is possible that the small scale, short-time regimen dependencies were mainly sequential interdependencies (e.g. administration of different medications as sequential steps), while the organisational dependencies were also relational or pooled, and that sequential interdependencies are easier to automate in workflow management systems.

Both ultra-safe and HRO strategies were used to address chemotherapy risks and interdependencies. CPOE automation supported application of ultra-safe strategies in chemotherapy such as compliance with protocols’ rules and ‘no-go’ contexts for proceeding with chemotherapy. CPOE support mechanisms for an ultra-safe model of care were both ‘hard’ (automation) and ‘soft’ (information availability/disambiguation). However, prescribing regimens with CPOE was difficult when protocol templates were not readily available; it was difficult to track medication data across screens, and a summary display of the whole regimen (showing protocol variations, if any) was not available; automated time-stamps of medication administration were not always accurate, with repercussions for subsequent time-dependent doses. Therefore, in addition to situational awareness at the level of the patient [38] and awareness of the organisation’s teams and services [27], clinicians had to maintain awareness of the technology (‘CPOE awareness’), its limitations and consequences. We propose a definition of CPOE awareness as knowledge of how the technology works, how to make it work, and the effects it produces, and acting on this knowledge.

The finding that ‘CPOE awareness’ was needed in this setting is consistent with other CPOE chemotherapy implementations that required significant investment of time and resources to enable clinicians to gain necessary familiarity with all the nuances of CPOE workflows [19]. Our finding that CPOE automation supported application of ultra-safe strategies in chemotherapy is in line with existing literature that shows how CPOE may improve compliance with chemotherapy protocols [17]. However, prescribed regimens are subject to change and in need of adaptation, also due to application of ultra-safe rules (typically, waiting for patient recovery before proceeding with treatment). In the late 1990s, medical sociologists Timmermans and Berg explained how standardisation of patient care with (paper-based) oncology protocols was achieved through clinicians’ ‘active (*not* mindless) support [...] to maintain the protocol’s trajectory on course’ [40]. In their account, adaptation was an enabler, rather than opposer, to standardisation. We posit that active (not mindless) adaptation requires situational awareness (knowledge of ‘*where is the patient at’* in relation to a prescribed regimen and underlying protocol), which must be facilitated by CPOE displaying interdependent elements of regimens over different time horizons (in chemotherapy, known as ‘roadmaps’ [12]). This is also supported by recent research suggesting that chemotherapy errors with CPOE may be prevented with knowledge of a patient’s chemotherapy history and in-depth knowledge of protocols [41]. Nurses’ and/or pharmacists’ routine checks of CPOE orders against protocols [22, 24] – also reported in the unit we studied - are one way to address CPOE limitations by automatically monitoring variations to protocols, and may also be facilitated by the availability of electronic roadmap displays.

Strengths of this study include multiple sources of data - interviews and incident reports - and analysis informed by theory. A limitation is that we did not observe the activities described. Instead, we relied on participants as ‘their own ethnographers’ [42]. We had limited nurse participation. We identified ultra-safe and HRO strategies; we did not find any strategy from the ultra-adaptive safety model possibly because we did not observe activities. Patient safety incident reports were brief and provided only limited information; incidents related to multiple aspects of patient care, not exclusively chemotherapy. We took a qualitative approach to incident analysis given the limitations of analysing these quantitatively; reported incidents are likely to represent only a fraction of the incidents that occur and thus numbers of incidents cannot be relied on to establish frequency [43]. The CPOE system investigated was a general system adapted for chemotherapy, rather than a bespoke chemotherapy system; it is not known whether the findings generalise across other organisations and other systems.

Ultra-safe services ‘are highly standardized and rely heavily on automation and information technology’ [3]. Implementation of CPOE in chemotherapy appears to be a move towards ultra-safe, but our findings suggest that CPOE design must be improved. In such a complex and high-risk setting, CPOE design should facilitate clinicians’ decision-making processes, rather than add difficulties. Lessons can be learned for design of chemotherapy CPOE that better supports the management of interdependencies in regimens and workflows [21]. This might include affording a variety of visualisation displays over different time horizons and capturing more accurate timestamps of activities, tracking protocol variations and cumulative effects over time. CPOE implementations also need to support learning processes for clinicians to gain the awareness needed to use CPOE systems safely. For this, sufficient enabling resources (e.g. staff/patient ratio, CPOE training and assistance, quiet time for prescribing) must be in place at the point of roll-out and maintained throughout staff turnover [19].

To our knowledge, this is the first study to apply Vincent and Amalberti’s models of safety and related risk strategies [3] to evaluate a technology implementation in a healthcare setting. The study has been able to identify mechanisms by which CPOEs can mitigate or exacerbate medication safety risks.

Patients and families may have different priorities or values about medication safety and compliance with rules, that may vary during the long period of chemotherapy treatments. Future research in paediatric oncology should investigate whether/how patients and families’ priorities or values change the way clinicians use the CPOE for chemotherapy regimens, and how they approach the different time frames: the ‘here and now’, ‘whole treatment’ and ‘life-long’. Further research with CPOE for chemotherapy should also investigate time dependencies in regimens in more detail – including how nurses manage administration times between doses and automated timestamping of activities.

## Conclusions

CPOE appears to affect the ‘mix’ of risk strategies in place in an oncology unit. It can drive ultra-safe models of safety and protocol mandated care, but operating ultra-safe strategies embedded in the CPOE and staying on protocol also requires HRO strategies including ‘sensitivity to operations’ in CPOE use. CPOE implementations need to support the processes of learning required for clinicians to gain such collective awareness, and shortcomings in CPOE design must be addressed for it to fully contribute to the ultra-safe.

## Supplementary information

**Additional file 1: Appendix 1:** Interview guide.

**Additional file 2.** Incident analysis - methods and summary of findings.

## Data Availability

The datasets generated and/or analysed during the current study are not publicly available. The hospital has not consented to the sharing of patient safety incidents data beyond the project team. Research participants did not consent to interview transcripts being made publicly available.

## Abbreviations

CPOE: Computerized provider order entry
HRO: High-reliability organisations
