# Supplementary material for: Electronic ordering and the management of treatment interdependencies: a qualitative study of paediatric chemotherapy
Source: BMC Med Inform Decis Mak. 2020 Aug 14;20:193. doi: 10.1186/s12911-020-01212-z (PMC7427723; doi:10.1186/s12911-020-01212-z)
Supplement: Supplementary file 1 — Additional file 1: Appendix 1: Interview guide. [file 12911_2020_1212_MOESM1_ESM.pdf]

Appendix 1: Interview guide

Depending on the time available, only a limited set of questions may be possible – these key questions have been signposted (*).

Opening	Introductions, confirm consent to recording, remind participant of the aim of the research and answer any questions (*)
Background	Participant role and experience with oncology medicines –<br>To start with, could you tell me about your role in the oncology ward?<br>Do you [prescribe][review][prepare and administer] medications? (*)<br>Is there a standard process for managing medications in the ward? What would be the typical way of [deciding on medications] for a patient?
Awareness of interdependencies	*Introduce the questions by explaining what we mean by interdependencies – e.g. oncology medications differ from other medications, with prescriptions set in terms of cycles, repeated over a period; scheduling of medications must be sequenced correctly, you need to monitor patient response, and then link orders to correct start time. There may be organisational constraints to keep in mind – such as giving enough time to pharmacy to prepare the medications -, or other aspects of the process that depend on 'something else' being completed. We call these complexities* interdependencies *in the medication management process.*<br>Are there interdependencies in existing workflows you should take into account when you [prescribe][review][prepare and administer] medications for a patient? (*)<br>Are [these interdependencies] potentially problematic, or are they easy to take into account?<br>How easy or difficult is it to maintain awareness of the overall process over time? Do paper tools or technology help? (*)
CPOE potential for transparency/ opacity over interdependencies	Compared to the way medications were managed before CPOE implementation, do you think the system provides more or less *transparency* over interdependent parts of the medications process over time? (*)<br>Could you give an example?
Potential for errors	Do you know of any incidents involving 'interdependencies' or awareness of the overall process – such as errors with scheduling or double prescriptions? Could you tell me about it? What do you think were the contributing factors?<br>Was this an error or a near-miss? [If it were a near-miss, ask how they realised the error, do they remember noticing that something was wrong]
Closing	Thanks to the participant and ask if they have any questions. (*)
