# Supplementary material for: Electronic ordering and the management of treatment interdependencies: a qualitative study of paediatric chemotherapy
Source: BMC Med Inform Decis Mak. 2020 Aug 14;20:193. doi: 10.1186/s12911-020-01212-z (PMC7427723; doi:10.1186/s12911-020-01212-z)
Supplement: Supplementary file 2 — Additional file 2. Incident analysis - methods and summary of findings. [file 12911_2020_1212_MOESM2_ESM.docx]

# Incident analysis: methods and summary of findings

We received from the hospital a dataset of 827 incidents voluntarily reported in oncology over a period of 18 months from initial CPOE roll out (15 August 2016 to 15 February 2018). In a previously published paper (1) we provide an in-depth description of the incidents, including the number of incidents that were medication‐related and CPOE‐related, and the distribution of incidents across the medication process (e.g. prescribing, administration). The aim of the first paper (1) was to identify medication safety issues related to use of the CPOE and contribution to incidents of CPOE design, configuration or use. We used an established classification scheme for this aim (2), applied to the incidents in a spreadsheet. Findings from our first analysis suggested the management of interdependencies (e.g. the automation of them) as an important theme. The present paper aims to examine interdependencies (in chemotherapy treatments) in greater depth, focusing on risk strategies to deal with them. To this aim, we took a qualitative inductive approach to the analysis of the dataset of incidents, supported by qualitative data analysis software NVivo. We explain below our methods.

## Methods for analysis

The analysis was carried out by one researcher (VL) and discussed with co-investigators.

As background for the analysis, we assessed the distribution of the incidents in the dataset in terms of incidents locations, patients and severity of incidents, to complement descriptive analysis previously carried out (1). We also noted that the description of the incident was often very brief, and the field *contributing factors* often empty. To confirm our sense of the limited nature of these accounts we counted in Excel the number of words in the field *incident description*.

We then carried out a qualitative content analysis of the free text fields of the 827 patient safety incidents.
We copied the fields *incident description* and *contributing factors* for all incidents from the dataset in a text file we imported into qualitative data analysis software (Nvivo). We used this to code each incident in the dataset with categories/themes. We took both a conventional (inductive) content analysis approach, with categories derived from the data, and a directed approach - our objectives providing higher level categories.

## Summary of findings

Not all incidents concerned direct patient care (e.g. some reported discrepancies in controlled drugs counts). When children were concerned, their age was varied – from babies to adolescents. Most incidents were reported under the *paediatric oncology* service; however, the dataset also contained incidents reported about oncology patients under other services, or a combination of services (Table A below), showing how the boundaries of patient care for patients in the unit are often ‘fluid’. Most incidents were reported in less than 43 words - the longest report being of 335 words
(Figure A). Not all incidents were about medications, as we report in (1); often we could not tell whether the incident related to chemotherapy treatments.

As we also report in (1), the hospital reporting system allows for four Severity Assessment Code (SAC) scores (1 to 4). This indicates the severity of any potential or actual consequence and the likelihood of the incident reoccurring (with higher scores indicating less severity). Not all 827 incidents were assigned SAC scores; among those that were rated for severity either by the reporting person (Initial SAC) and/or a manager (Actual SAC), incidents had respective averages 3.3 (n=433) and 3.4 (n=628).(1)

Table A. Services reported as involved in the incidents

| **Incident report field: ‘*Specific Service’*** | | |
| --- | --- | --- |
| 1. Anaesthetics; Paediatric Oncology | 1. Intensive care | 1. Paediatric Oncology; Other; Specify (Blood bank) |
| 1. Anaesthetics; Paediatric Oncology; Pain management | 1. Medical Oncology | 1. Paediatric Oncology; Other; Specify (Hospital School) |
| 1. Clinical psychology; Paediatric Oncology | 1. Medicine - General; Medical Oncology | 1. Paediatric Oncology; Paediatric Surgery |
| 1. Corporate Services | 1. Neonatology; Paediatric Oncology | 1. Paediatric Oncology; Paediatric Surgery; PADP |
| 1. Dental - Oral Health; Paediatric Oncology | 1. Neurology; Paediatric Oncology; Neurosurgery | 1. Paediatric Oncology; Pain management |
| 1. Emergency medicine; Neurology; Paediatric Oncology; Neurosurgery | 1. Other; Specify (Oncology (BMT)) | 1. Paediatric Oncology; Pain management; Intensive care |
| 1. Emergency medicine; Paediatric Oncology | 1. Paediatric Oncology | 1. Paediatric Oncology; Palliative Care |
| 1. Haematology; Paediatric Oncology | 1. Paediatric Oncology; Intensive Care | 1. Paediatric Oncology; Pharmacy |
| 1. Haematology; Paediatric Oncology; Pathology | 1. Paediatric Oncology; Ambulance Administrative services | 1. Paediatric Oncology; Transplant Services |


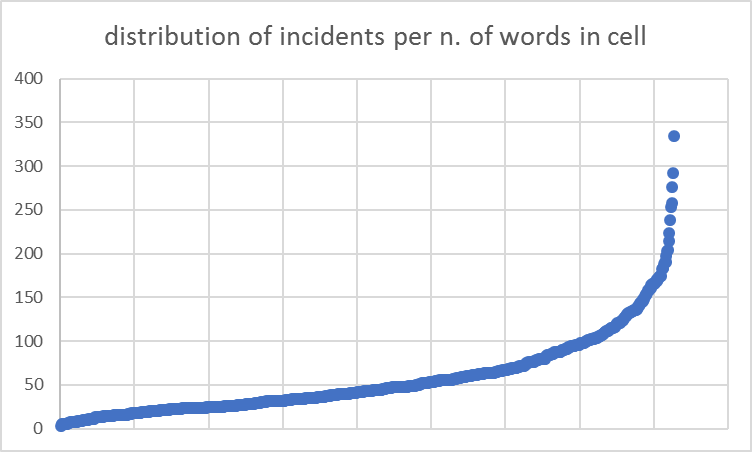


Figure A. Distribution of incidents per number of words in the field 'incident description'

Our analysis in NVivo derived 93 codes over four mains overarching categories –*interdependencies* (-in relation to...), *sensitivity to operations*, (issues with) *risk strategies* and (issues with) the *CPOE* (Figure B). Issues with CPOE comprised all incidents where the CPOE was mentioned. Codes were both descriptive of the reality of patient care (e.g. services involved in the incident) and descriptive of the consequences of the incidents (for example in terms of delays or need for rescheduling). The categories and codes were not exclusive (e.g. an incident coded under ‘CPOE – issues with/time and dates’ could also be coded under ‘Interdependencies/Organisation of Clinical Activities’. Among the (issues with) risk strategies, we found CPOE safety mechanisms, such as the required ‘ready for chemo tick’. Thus, the CPOE appeared to be both a mechanism for safety and a contributing factor for processes not going as desired. The CPOE was not the only technology utilised as a safety mechanism (others were smart pumps and ventilators alarms).


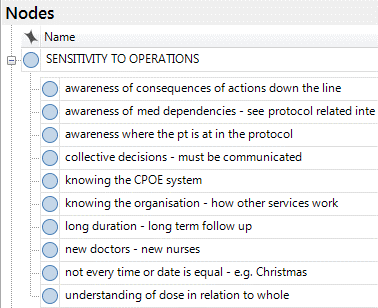

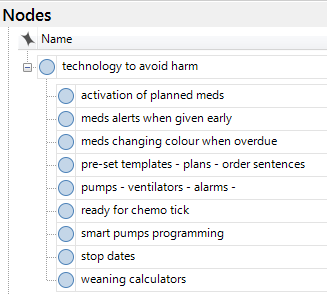

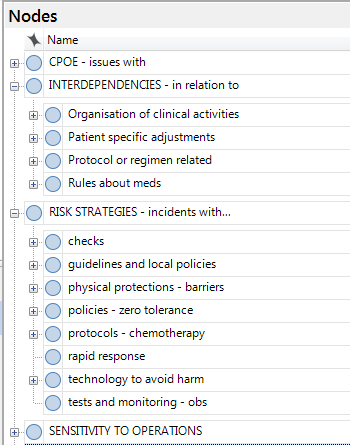


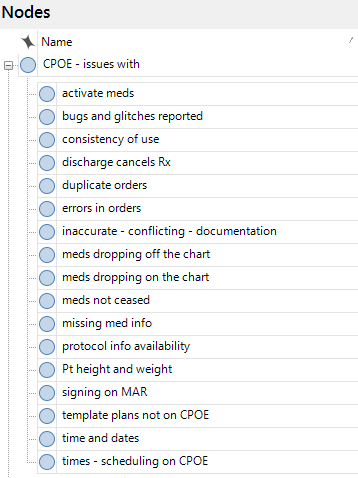


Figure B. Examples of analytical codes and higher level categories (nodes) in Nvivo

The time dimension was especially apparent across the incidents. One or more codes about ‘time’ were present in each of the four main categories. For example, not only were some interdependencies *sequential in time*, but also doses were cumulative *over time*, medications must not be given *at the same time*, IVs must be given at certain *speed* (vol / time), there were *time frames* (‘deadlines’, expiry dates) by which certain tasks must have been done or information must have been provided, and consequences in terms of *delays*.

We found some incidents suggested clinicians’ awareness of the risks associated with interdependencies in a patient treatment. For example, we found awareness of interdependency between medications, or between times of medications; awareness of hospital services across areas and the challenge of organising the services to match times of dependent tasks without delays; and awareness of consequences for others of own (or others’) actions – for example the risks of missing a midnight dose after discharge.

References

1. Lichtner V, Baysari M, Gates P, Dalla-Pozza L, Westbrook JI. Medication safety incidents in paediatric oncology after electronic medication management system implementation. European Journal of Cancer Care. 2019;28(6):e13152.

2. Sittig DF, Classen DC, Singh H. Patient safety goals for the proposed Federal Health Information Technology Safety Center. Journal of the American Medical Informatics Association : JAMIA. 2015;22(2):472-8.
